# P-2190. Letermovir use for CMV prophylaxis in lung transplant recipients : A single center experience

**DOI:** 10.1093/ofid/ofaf695.2353

**Published:** 2026-01-11

**Authors:** Mario A Torres, Joanna K Nelson, Koray Demir, Alex Zimmet, Aruna Subramanian, Thomas Dieringer

**Affiliations:** Stanford University , Menlo Park , CA; Stanford University, Stanford, CA; Stanford University, Stanford, CA; Stanford Healthcare, Stanford University School of Medicine, Palo Alto, California; Stanford University, Stanford, CA; Stanford University, Stanford, CA

## Abstract

**Background:**

Cytomegalovirus (CMV) is a significant complication in lung transplant recipients (LTRs). While valganciclovir is commonly used for prophylaxis, its use is often limited by toxicity. Letermovir (LET) has shown promise in renal and hematopoietic stem cell transplant recipients, but data in LTRs are limited. Here, we report on the safety and effectiveness of letermovir for CMV prophylaxis in lung transplant recipients.

**Table 1:**
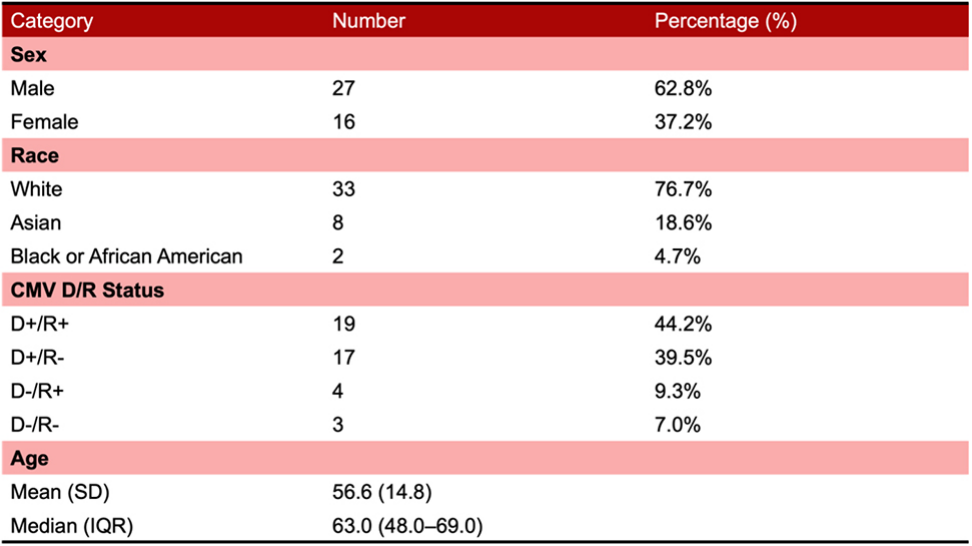
Demographics

**Figure 1:**
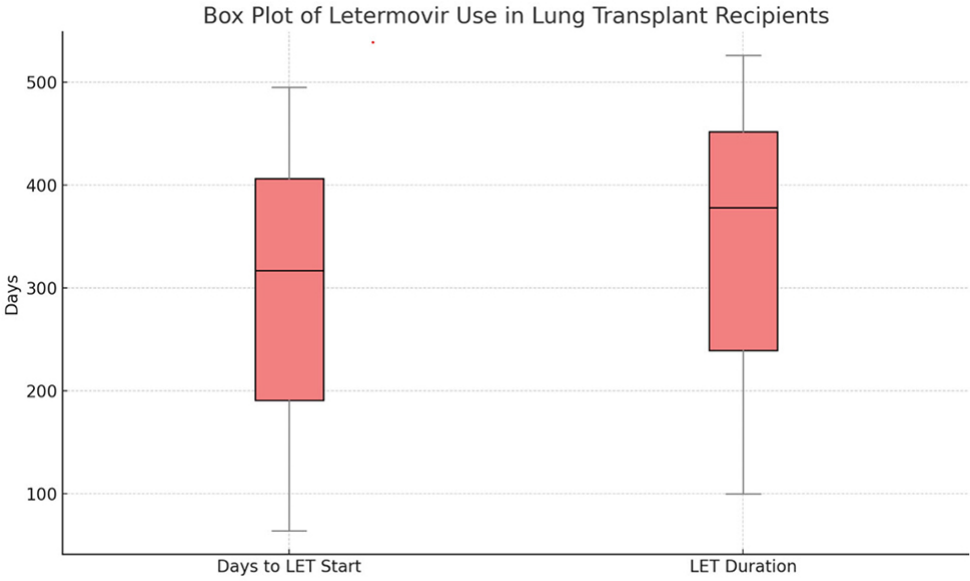
Box Plot

**Methods:**

In this single-center, retrospective study, we evaluated the safety and effectiveness of LET for primary and secondary CMV prophylaxis in lung transplant recipients at Stanford Health Care between January 2017 and December 2024. Data was collected through April 2025. Data extracted from electronic medical records included CMV serostatus, indication and timing of LET initiation/discontinuation, CMV viral load, immunosuppression regimens, and clinical outcomes. Descriptive statistics were used for analysis.

**Table 2:**
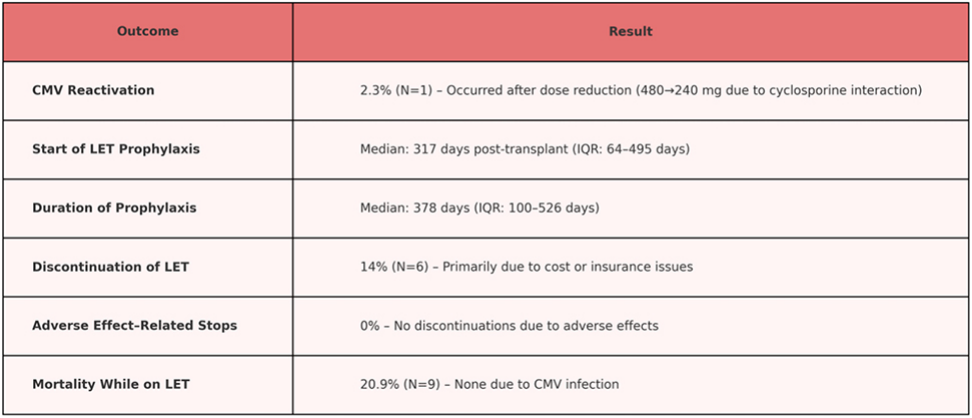

**Results:**

A total of 43 lung transplant recipients who received letermovir prophylaxis were included. The median age was 56 years, and 62.8% were male. CMV serostatus was D+/R− in 39.5%, D+/R+ in 44.2%, D−/R+ in 9.3%, and D−/R− in 7.0% (Table 1). LET was started in 83.3% (N=35) due to valganciclovir-associated leukopenia. The standard dose of letermovir was 480 mg.

CMV reactivation was defined as CMV DNA detected above institutional lab threshold ( >135 copies/mL) and occurred in 2.3% (N=1), attributed to a dose reduction from 480 mg to 240 mg daily, due to drug interaction with cyclosporine. LET was started at a median of 317 days post-transplant (IQR 64-495), with median duration of prophylaxis being 378 days (IQR 100-526) (Figure 1). LET was stopped in 14% (N=6), primarily due to cost or insurance issues. No discontinuations were due to adverse effects.

Mortality while on LET occurred in 20.9% (N=9); however, none of the deaths were attributed to CMV infection. (Table 2)

**Conclusion:**

Letermovir prophylaxis was associated with a low incidence of clinically significant CMV reactivation or infection in LTRs, along with a favorable safety profile. These findings support its potential as a viable alternative to traditional CMV prophylaxis in this population. However, further studies are warranted to confirm its long-term effectiveness and broader applicability.

**Disclosures:**

Aruna Subramanian, MD, Gilead: Grant/Research Support|Moderna: Grant/Research Support

